# Wearable Arrhythmia Detection: Moving from your Watch to your Shirt?

**DOI:** 10.1007/s11886-026-02445-6

**Published:** 2026-11-03

**Authors:** Janna-Luisa Beermann, David Duncker

**Affiliations:** Hannover Heart Rhythm Center, Department of Cardiology and Angiology, Hannover Medical School, Carl-Neuberg-Str, 1, 30625 Hannover, Germany

**Keywords:** Wearables, Arrhythmia detection, Smart garments, Atrial fibrillation, Smart watch, Artificial intelligence

## Abstract

**Purpose of Review:**

Digital devices play an increasingly important role in the detection and screening of cardiac arrhythmias. Atrial fibrillation (AF), the most common arrhythmia, is associated with an increased risk of stroke, heart failure, and mortality. This review summarises the current evidence on wearable devices for screening and diagnosing atrial fibrillation.

**Recent findings:**

Numerous studies have demonstrated the value of wearables for AF detection and diagnosis. In addition, artificial intelligence enhances automated rhythm analysis and signal processing, supporting the increasing clinical implementation of digital technologies in outpatient care.

**Summary:**

Single-lead ECG devices provide the strongest evidence for AF detection, with smartwatches representing one of the most extensively investigated wearable technology. Limitations include motion artefacts and predominantly semi-continuous monitoring. Future research should focus on integrating artificial intelligence to improve signal analysis and artefact reduction, and on evaluating the impact of wearable-based strategies on clinically relevant outcomes, including stroke and heart failure.

## Introduction

Digital devices for cardiac rhythm monitoring play a key role in the detection and screening of cardiac arrhythmias [1]. A wide variety of digital devices are available. In addition to smartwatches, smart rings, ECG patches and smart textiles are also becoming increasingly important [2].

Utilisation of wearable ECG monitoring goes beyond the documentation of symptomatic cardiac arrhythmias. It can also be used for the screening of asymptomatic arrhythmias and for the quantification of the arrhythmia burden [3–5].

Atrial fibrillation (AF) is the most prevalent persistent cardiac arrhythmia and is associated with an elevated risk of stroke, heart failure and mortality. Early detection and the initiation of treatment can have a positive impact on comorbidities [6]. Conventional methods of long-term ECG monitoring are limited by the restricted monitoring time. The diagnosis of paroxysmal and asymptomatic AF presents a particular challenge. Through automatic and (semi-)continuous rhythm monitoring, wearables play an important role in screening, diagnosis and the initiation of treatment. In recognition of this advance, current guidelines recommend the use of wearables for the screening and diagnosis of AF [7–9]. They can also be used for long-term monitoring to detect the recurrence of AF or other cardiac arrhythmias. This allows the burden of AF to be assessed in relation to symptoms following ablation, electrical cardioversion or antiarrhythmic drug therapy [4]. Furthermore, wearables showed a positive impact on medication adherence, patient satisfaction and quality of life [10].

This review focuses on the detection of arrhythmias using smartwatches or smart garments, especially diagnosing and screening for AF.

### Digital Health Technologies

The classification of wearable heart rate monitoring technologies can be categorised into two distinct systems: electrocardiography (ECG) and photoplethysmography (PPG) [1].

The underlying principle of PPG technology is the optical detection of changes in blood volume via light absorption [11]. The light-emitting diodes project light of a specific wavelength into the tissue, which is reflected and detected by the device. The changes in light absorption, which are dependent on pulsatile blood flow, are used to generate a pulse waveform.

This can facilitate the identification of arrhythmias, particularly AF [12, 13]. PPG-based systems are suitable for the assessment of heart rate, heart rhythm and arrhythmia burden [4, 14]. Several studies have shown that PPG-based wearables can be used to detect AF [14, 15]. However, signal quality depends on several factors, including skin colour, skin texture and movement intensity [16]. In addition to smartwatches, PPG-based wearables include smartphone applications using finger or facial recognition, as well as fitness trackers​ [17]. Nevertheless, ECG confirmation remains necessary for a definitive diagnosis [7].

ECG-based heart rhythm monitoring remains essential for the diagnosis of cardiac arrhythmias. The number of leads used in wearable ECG devices can vary between systems. The validity of the single-lead ECG in the detection of cardiac arrhythmias has been demonstrated [18, 19]. Initially, ECG recordings could only be made using external hardware. Nowadays, the use of integrated electrodes allows for the recording of high-quality single-lead ECGs [20].

### Clinical Use

**Fig. 1:**
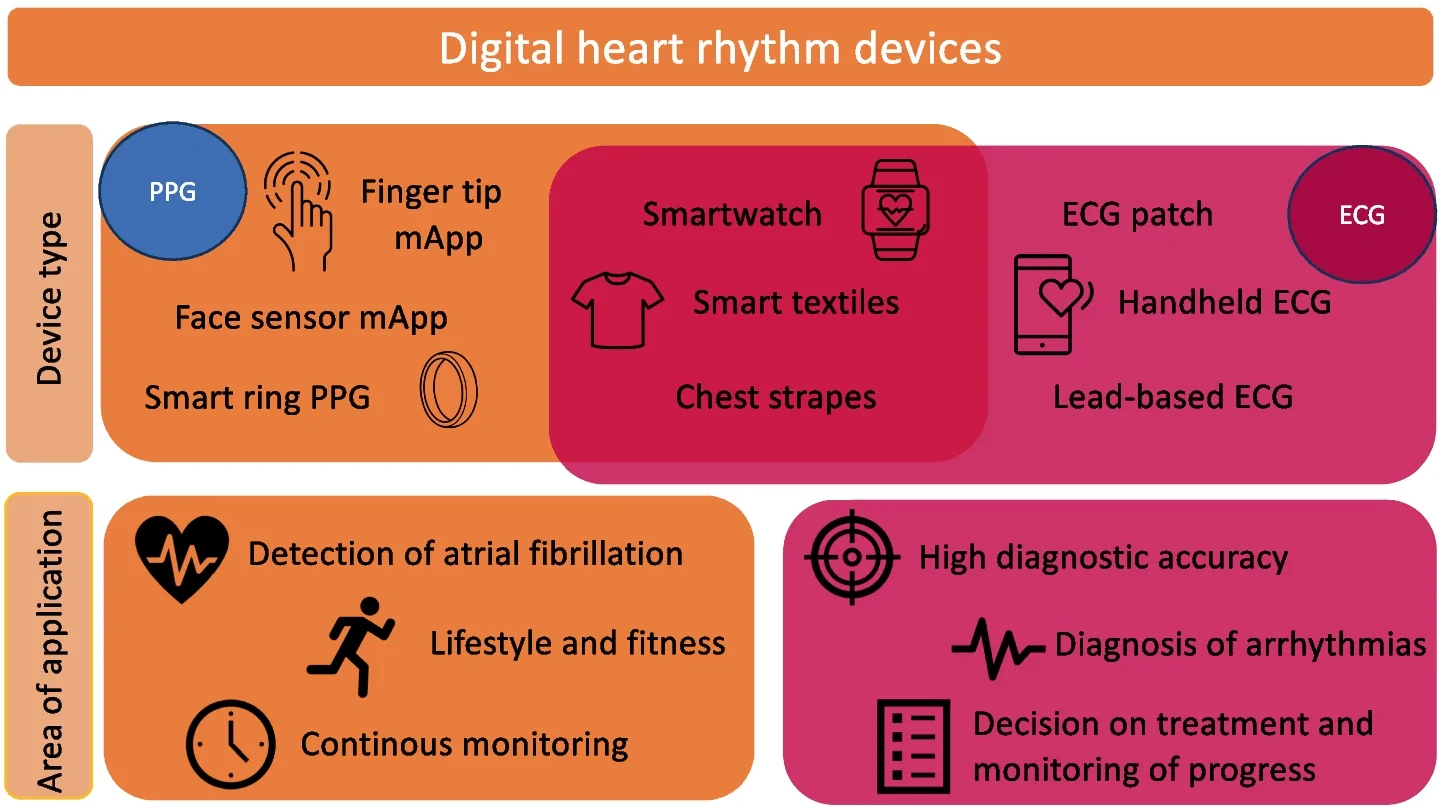
Overview of digital heart rhythm devices for the clinical use. The devices have been categorised according to whether they monitor heart rhythm using PPG or ECG. Some devices use both ECG and PPG. *ECG* electrocardiography, *mApp* mobile App, *PPG* photoplethysmography

The 12-lead ECG remains the gold standard for ECG diagnostics. However, the challenge remains in terms of its availability and the provision of continuous ECG monitoring, particularly for the detection of asymptomatic or paroxysmal cardiac arrhythmias. Various devices are available for this purpose (Fig. 1).

ECG-based wearable devices comprise a range of categories, each with its own technical specifications and areas of application. Depending on the model, portable Holter ECG systems can record via integrated electrodes – for example, through finger contact – or via additional skin electrodes on the chest and limbs [17, 21].

An increasingly established alternative is represented by ECG patches. Due to their ease of use and the ability to monitor rhythm continuously over a period of five to 30 days, they achieve high patient adherence [22]. Another development in this technology are smart garments, which have integrated electrodes that enable continuous multi-channel ECG recording [22].

Smartwatches, depending on their technical design, can carry out rhythm recording via photoplethysmography (PPG) on the wrist or via integrated electrodes to record a 30-s single-lead ECG [23]. This can be displayed in real time on the device or in the accompanying smartphone app. Consequently, smartwatches have become particularly important for screening and detecting AF. Modern algorithms can already detect AF automatically [24, 25], although this still requires medical validation using an electrocardiogram.

### Smartwatch in the Diagnosis of Atrial Fibrillation

The use of smartwatch for the detection of cardiac arrhythmias has been the subject of numerous studies [12, 14, 24]. These studies demonstrated the clinical applicability of PPG-based consumer wearables for screening purposes in relation to AF. ​An overview of selected trials using smartwatches or smartphone-based PPG is shown in Table 1.

**Table 1:** Overview of selected trials using smartwatches and smartphone-based PPG for atrial fibrillation detection

Study	Year	Design	Technology	Clinical Relevance
Apple Heart Study [24]	2019	Prospective virtual study	Apple Watch (PPG) + ECG patch confirmation	First large-scale smartwatch study establishing PPG-based AF screening
MAFA II -Study [12]	2019	Prospective screening study	Huawei smart devices (PPG)	Validated PPG-based screening in a large Asian population
Fitbit Heart Study [25]	2022	Prospective virtual study	Fitbit smartwatch/fitness tracker (PPG)	Confirmed the performance of an AI-assisted PPG algorithm
eBRAVE-AF Trial [26]	2022	Randomized controlled trial	Smartphone-based PPG (FibriCheck)	First RCT demonstrating the clinical benefit of digital AF screening
TeleCheck-AF [27]	2021	Multicentre implementation study	Smartphone-based PPG (FibriCheck)	Supported implementation of digital rhythm monitoring in clinical practice
SMARTBEATS ALGO [28]	2025	External validation study	Smartphone-based PPG + AI	Demonstrated the potential of AI-assisted PPG algorithms for AF detection

*AF* Atrial fibrillation, *AI* Artificial intelligence, *ECG* Electrocardiography, *PPG* Photoplethysmography, *RCT* Randomized controlled trial

The eBRAVE-AF trial represented an important milestone for the clinical implementation of digital screening strategies [26]. The study is a digital, randomized, prospective, open-label, cross-over study investigated whether smartphone-based PPG screening could improve the detection of previously undiagnosed AF​ compared with standard care. They examined PPG-based screening methods exclusively. The study demonstrated a significantly higher detection rate of AF in the screening group. Although, the focus was not on clinical endpoints such as the reduction of AF-associated complications or the improvement of long-term prognosis [26].

Recent meta-analyses have confirmed the high diagnostic performance of the smartwatch ECG for the detection of AF [27]. Furthermore, diagnostic accuracy has improved. Results can be displayed immediately via various apps and sent to treating healthcare professionals. The continuous rhythm monitoring capabilities of smartwatches make them particularly suitable for the screening for paroxysmal AF [28, 29]. Nowadays, modern smartwatches utilise a combination of both technologies. (Semi-)Continuous PPG monitoring alongside an event-driven ECG is enabled, for example when an irregular pulse is detected [23, 30]. In the randomized EQUAL trial, 6-month smartwatch-based monitoring significantly increased the detection of previously undiagnosed atrial fibrillation in older individuals at elevated stroke risk compared with standard care (9.6% vs. 2.3%). This highlighting the potential of wearable-based screening when integrated into a structured telemonitoring workflow [31].

Medical interpretation further enhances diagnostic accuracy in comparison with automated device analysis [28]. Consequently, the current guidelines categorise wearables predominantly as screening instruments [7, 8]. However, a definitive diagnosis still requires confirmation by a physician based on an ECG.

The diagnostic capabilities of smartwatches are subject to some limitations. In the context of physical activity, the presence of motion artefacts has been demonstrated the effect on signal quality, resulting in false-positive or false-negative results [27]. Furthermore, ECG recording still requires active user interaction. Studies on the automated detection of AF with subsequent notification of the patient have shown a lower detection rate compared with implantable loop recorders [32].

In addition, the presence of pre-existing ECG abnormalities has been demonstrated to compromise the precision of automated classification algorithms employed in the context of single-lead ECGs [33]. These limitations underscore the necessity for careful patient selection and medical validation of abnormal findings.

### Detection of Arrhythmias other-than Atrial Fibrillation

Apart from AF, these devices demonstrated capability in detecting various other arrhythmias, such as second and third degree atrioventricular block, atrial flutter, atrial tachycardia, supraventricular tachycardia, atrioventricular nodal re-entrant tachycardia, atrioventricular re-entrant tachycardia, or ventricular tachycardia [34]. A major limitation of PPG-based rhythm analysis is that supraventricular and ventricular tachycardias is difficult to reliably distinguished on the basis of the PPG signal. Early findings have shown that machine learning algorithms trained using PPG data recorded during electrophysiological examinations can distinguish between supraventricular and ventricular tachycardias on the basis of PPG signals [35]. Consequently, only a few algorithms are currently available for the reliable differentiation of various tachyarrhythmias. Furthermore, continuous rhythm monitoring is necessary, particularly in cases of syncope or haemodynamically significant tachycardias. In these situations, user-initiated recording is generally not possible [36].

### Smart Garments for Arrhythmia Detection

Smart garments are defined as textile wearables with integrated electrodes that enable continuous electrocardiographic monitoring [37]. In addition, multi-channel ECG analysis can be carried out using leads recorded simultaneously [38], allowing the detection of cardiac arrhythmias [37, 39]. A small study compared the measurement of heart rate variability using a smart shirt and a 12-lead ECG at various levels of exertion on a bicycle ergometer. It has been demonstrated high measurement accuracy even during physical activity, although signal quality decreased as exercise intensity increased [40] Overall, smart garments are still undergoing technical and clinical validation for clinical use.

To date, the deployment of smart garments has been primarily focused in non-medical contexts, with a notable emphasis on their utilisation by athletes for training purposes and lifestyle monitoring, while medically validated systems are also available. The system that has been most extensively studied to date is a smart shirt with integrated electrodes enabling continuous single-lead ECG recording (Hexoskin Medical System, California, USA). In paediatric patients with heart conditions, the diagnostic accuracy of the test was comparable to that of a Holter ECG [41]. Another study showed that the heart rate measurements from a textile-based 12-lead ECG were highly consistent with those from a Holter ECG [42]. A textile ECG-based machine-learning algorithm enabled highly accurate detection and quantification of AF burden during long-term monitoring, supporting its potential as a practical alternative to conventional Holter monitoring [43].

The ECG signal quality during periods of high physical exertion remains challenging [40]. It has been shown that high recording quality can be achieved primarily during periods of rest, as well as whilst sitting or standing and during moderate physical activity [44, 45]. Smart garments therefore expand the possibilities and appear particularly suitable for screening, remote monitoring and the detection of paroxysmal arrhythmias.

### Arrhythmia Management with Wearables

During the COVID-19 pandemic, many centres gained experience in the use of on-demand digital devices [46]. Digital wearables are increasingly being used to follow up with patients after they have undergone catheter ablation or electrical cardioversion, especially for detection of arrhythmia recurrence and the quantification of AF burden following catheter ablation [47, 48]. Continuous PPG-based rhythm monitoring demonstrated comparable diagnostic accuracy in detecting AF to that of an implantable loop recorder (ILR) [49].

Furthermore, digital follow-up using a smartphone-based PPG application was not inferior to conventional follow-up over a period of 3 to 12 months following catheter ablation [50]. Digital monitoring also achieved higher detection rates of arrhythmia recurrence [51]. Patient adherence was high even when symptom burden was low [48]. Both smartwatch-based single-channel ECGs and ECG patches demonstrated good results for rhythm monitoring during follow-up after catheter ablation [50, 52]. In a smale-scale study a continuous 2-week monitoring with a smart shirt ECG detected a greater extent of AF recurrence than 24-h Holter monitoring, despite a lower ECG acquisition rate [53]. By transmitting ECG data via digital devices, targeted cardioversion can be planned in cases of new-onset AF only if AF persists [54]. A prospective multicentre study demonstrated that digital devices can support the management of patients prior to planned cardioversion by detecting spontaneous rhythm conversions, thereby avoiding unnecessary hospital admissions and transoesophageal echocardiograms [55]. Additionally, a machine learning algorithm for the automated analysis of smartphone-based PPG recordings in patients following electrical cardioversion in an outpatient setting was found to be highly accurate in detecting AF [56].

It remains unclear whether wearable monitoring can lead to earlier therapeutic intervention, such as repeat cardioversion, re-ablation, or adjustment of drug therapy, and whether this impacts clinically relevant endpoints, such as stroke, hospitalisation, or arrhythmia recurrence, compared with standardised follow-up.

### Patient Engagement Perspective

The use of digital devices in the monitoring of cardiac arrhythmias has an impact on quality of life, self-management of the condition and patients’ understanding of their condition [57]. Several studies also show a high level of patient acceptance and adherence to drug-based or invasive treatment, particularly due to the ease of use and the ability to monitor one’s own heart rhythm, which can help to improve symptom management [10, 58, 59].

### Perspectives in Arrhythmia Detection with Wearables

The development of digital wearables is ongoing, and the integration of artificial intelligence (AI) is becoming increasingly important for the detection and management of AF [29, 56, 60]. Current research focuses on developing and validating AI-based algorithms for detecting AF and on AI-assisted analysis of electrocardiographic data. AI-based algorithms can analyse pulse intervals and detect irregularities in the heart rhythm [16]. Moreover, several studies have shown that analysing ECG recordings in sinus rhythm with the help of AI can identify arrhythmogenic trigger factors and allow risk stratification with regard to the onset of AF​ [61, 62]. The use of AI enables the automated classification of AF, the suppression of motion artefacts, and improved differentiation between artefacts and pathological rhythm events. This helps to reduce false-positive and false-negative findings and improve the quality of signal processing. Furthermore, AI-supported quality control enables a standardised assessment of signal quality. In the future, these developments could also contribute to the optimisation of smart garments for the screening of cardiac arrhythmias, particularly in children or in the context of sports medicine [60]. The integration of AI and technological advancements has enabled modern smartwatches to facilitate the capture of single-lead ECG representing a substantial development in healthcare monitoring [63].

## Conclusion

Wearable devices have become established for detecting and monitoring cardiac arrhythmias. Smartwatches are supported by a large body of evidence and enable reliable screening for AF through the combination of PPG and single-lead ECG. Smart garments can facilitate long-term rhythm monitoring by providing continuous multi-channel ECG recordings. However, despite their high diagnostic accuracy, limitations remain, including motion artefacts, limited differentiation between complex arrhythmias and the need for ECG confirmation.

Integrating AI could lead to automated rhythm analysis, artefact reduction, and improved signal quality. Furthermore, AI-supported analysis of ECG and PPG signals could contribute to optimised remote digital pathways in the future.

## Key References

- Svennberg E, Merino JL, Andrade J, Anselmino M, Arbelo E, Boersma E, et al. Transforming atrial fibrillation management by targeting comorbidities and reducing atrial fibrillation burden: the 10th AFNET/EHRA consensus conference. Europace. 2025;27(12).
  - Personalized, safe, and effective rhythm-control strategies are needed to reduce AF burden and cardiovascular events through optimized drug therapy and advances in ablation.
- Zuo S, Zhou L, Feng H, Massad C, Khalil MA, Wang J, et al. Validation of Smartwatches Integrated With Photoplethysmography for Continuous Evaluation of Atrial Fibrillation Burden. JACC Clin Electrophysiol. 2026.
  - Smartwatches equipped with photoplethysmography (PPG) can measure atrial fibrillation (AF) burden continuously and with a high degree of accuracy, and thus represent a practical, non-invasive alternative to continuous ECG monitoring.

## Data Availability

No datasets were generated or analysed during the current study.

## Abbreviations

AF: Atrial fibrillation
AI: Artificial intelligence
ECG: Electrocardiography
ILR: Implantable Loop Recorder
ML: Machine Learning
PPG: Photoplethysmography
RCT: Randomized controlled trial
